# Involvement of GANP in B Cell Activation in T Cell-dependent Antigen Response

**DOI:** 10.1080/1044667031000137647

**Published:** 2002-09

**Authors:** Nobuo Sakaguchi, Satoru Fujimura, Kazuhiko Kuwahara

**Affiliations:** 1 Department of Immunology Graduate School of Medical Sciences Kumamoto University 1-1-1 Honjo Kumamoto 860-0811 Japan; 2 CREST Japan Science and Technology Corporation (JST) Japan; 3 PRESTO Japan Science and Technology Corporation (JST) Japan

## Abstract

Adaptive immunity is dependent on proliferation of antigen-driven B cells for clonal expansion in germinal centers (GCs) against T cell-dependent antigens (TD-Ag), accompanied with somatic hypermutation of variable-region gene and class switching of B cell antigen receptors. To study molecular mechanisms for B cell differentiation in GCs, we have identified and studied a 210 kDa GANP protein expressed in GC-B cells. GANP has domains for MCM3-binding and RNA-primase activities and is selectively up-regulated in centrocytes surrounded with follicular dendritic cells (FDCs) upon immunization with TD-Ag *in vivo* and in B cells stimulated with anti-CD40 monoclonal antibody *in vitro*, which suggested that GANP plays a certain important role in the maturation of immunoglobulin or selection of B cells in GC during the immune response to TD-Ag. Since this up-regulation has not been detected in T cells in GCs and in Concanavalin A-stimulated T cells *in vitro*, selective function of GANP molecule on B cell proliferation and differentiation might exist.


*Developmental Immunology*, September 2002, Vol. 9 (3), pp. 169–172

Taylor & Francis

Taylor & Francis Group

### Involvement of GANP in B Cell Activation in T Cell-dependent Antigen Response*

NOBUO SAKAGUCHI[a,b,†], SATORU FUJIMURA[a] and KAZUHIKO KUWAHARA[a,c]

[a]*Department of Immunology, Graduate School of Medical Sciences, Kumamoto University, 1-1-1, Honjo, Kumamoto 860-0811, Japan;* [b]*CREST, Japan Science and Technology Corporation (JST), Japan;* [c]*PRESTO, Japan Science and Technology Corporation (JST), Japan*

Adaptive immunity is dependent on proliferation of antigen-driven B cells for clonal expansion in germinal centers (GCs) against T cell-dependent antigens (TD-Ag), accompanied with somatic hypermutation of variable-region gene and class switching of B cell antigen receptors. To study molecular mechanisms for B cell differentiation in GCs, we have identified and studied a 210 kDa GANP protein expressed in GC-B cells. GANP has domains for MCM3-binding and RNA-primase activities and is selectively up-regulated in centrocytes surrounded with follicular dendritic cells (FDCs) upon immunization with TD-Ag *in vivo* and in B cells stimulated with anti-CD40 monoclonal antibody *in vitro*, which suggested that GANP plays a certain important role in the maturation of immunoglobulin or selection of B cells in GC during the immune response to TD-Ag. Since this up-regulation has not been detected in T cells in GCs and in Concanavalin A-stimulated T cells *in vitro*, selective function of GANP molecule on B cell proliferation and differentiation might exist.

*Keywords*: CD40; GCs; RNA-primase; TD-Ag

### INTRODUCTION

Antigen stimulation induces B cell activation for proliferation and differentiation in peripheral lymphoid organs (Rajewsky, 1996). In the immune response to soluble T cell-dependent antigens (TD-Ag), B cells gather in the follicular region, creating germinal centers (GCs), which are surrounded by activated Th cells. In GCs, Ag-driven B cells proliferate rapidly as centroblasts in the dark zone and arrest cell cycling as centrocytes in the light zone (MacLennan, 1994; Kolsoe, 1996). During the formation of GCs, B cells differentiate through various molecular processes undertaking altered expression of various surface molecules, variable-region (V-region) somatic hypermutation, and class switching of antibodies (Abs). It is thought that the differentiated B cells bearing high affinity B cell receptors (BCRs) are selected through the network of follicular dendritic cells (FDCs) presenting tiny amounts of exogenous Ags in GCs. As a selection mechanism, the expression levels of BCR and the co-stimulatory molecules are potentially important for the signal transduction leading to the death or survival of Ag-reactive B cells. To understand the molecular mechanism of B cell differentiation, we searched for molecules that are up-regulated or expressed selectively in GC-B cells by preparing monoclonal antibodies (mAbs) against the B cell lysate. A mAb identified a 210-kDa nuclear protein named GANP that is expressed in GC-B cells at the centroblast and centrocyte areas (Abe *et al.*, 2000; Kuwahara *et al.*, 2000). Using the mAb, a 6-kb cDNA was isolated and the expression of the mRNA was confirmed as GC-selective manner. GANP is a differentiation Ag that is expressed in the nucleus of GC-B cells upon immunization with TD-Ag.

### MATERIALS AND METHODS

### Mice

All mice were purchased from Charles River Japan (Yokohama, Japan) and maintained in the Center for Animal Resources and Development (CARD), under the regulation for Animals of Kumamoto University. Female and male C57BL/6 mice were immunized with various kinds of TD-Ag.

*Presented at the Proceedings of the 4th Germinal Center Conference, Groningen, The Netherlands, June 2002.

†Corresponding author. Address: Department of Immunology, Graduate School of Medical Sciences, Kumamoto University, 1-1-1, Honjo, Kumamoto 860-0811, Japan. Tel.: +81-96-373-5134. Fax: +81-96-373-5138. E-mail: nobusaka@kaiju.medic.kumamoto-u.ac.jp

ISSN 1044-6672 print © 2002 Taylor & Francis Ltd
DOI: 10.1080/1044667031000137647

### Immunization

Trinitrophenyl keyhole limpet hemocyanin (TNP-KLH; Biosearch Technologies, Novato, CA) (50 μg) emulsified with complete Freund's adjuvant was immunized into peritoneal cavity of C57BL/6 mice.

### Immunohistochemistry

After immunization, the lymphoid organs were obtained and frozen sections were prepared as described previously (Kuwahara *et al.*, 2000). The reagents for immunostaining such as biotin-labeled peanut agglutinin (PNA; Vector Laboratories, Burlingame, CA), peroxidase-conjugated streptavidin (Kirkegaard and Perry Laboratories, Gaithersburg, MD) and alkaline phosphatase-conjugated goat anti-rat IgG Ab (Southern Biotechnology Associates, Birmingham, AL) were purchased. The development was performed using Vector Blue kit (Vector) or 3-3′-diaminobenzidine tetrahydrochloride (Donjindo, Kumamoto, Japan).

### RESULTS

### Molecular Structure of GANP

The mouse GANP protein of 1971 amino acids showed a structural similarity with human Map80, that was identified as a molecule associated with the MCM3 component of origin (ori)-recognition complex by other investigators (Takei and Tsujimoto, 1998). Structural and genomic analysis demonstrated that GANP is a longer isoform generated presumably by an alternative RNA splicing of the same gene *ganp/Map80* allele on chromosome 21 in human (Abe *et al.*, 2000). GANP carries the MAP80 region at the carboxyl-terminal end and showed an association with MCM3, suggested that GANP is involved in regulation of DNA-replication in GC-B cells (Abe *et al.*, 2000; Kuwahara *et al.*, 2000) (Fig. 1). MCM3 is a component of ori-binding complex composed of six molecules as MCM-2, -3, -4, -5, -6 and -7. The complex is thought as a replication licensing factor that permits the cell division after the completion of appropriate DNA replication. The molecular function of MCM-complex had not been determined but a recent report demonstrated that MCM complex bear a helicase activity that is capable of unwinding the double strand DNA during G1 to S phase of cell cycle (Ishimi, 1997). Map80 also showed an acetylating activity on MCM3, which is presumably involved in the association with DNA strands (Takei *et al.*, 2001).

We found that GANP carries a region weakly homologous to RNA/DNA primase $\alpha$ of p49, which is a component of primase $\alpha$/DNA polymerase $\alpha$ (p49, p58, p70 and p180) (Waga and Stillman, 1998; Arezi and Kuchta, 2000). The primase activity is essential for the extension of lagging-strand synthesis, which requires a 20–30-nucleotide RNA primer for the initiation of DNA synthesis, leading to the discontinuous chain elongation in the opposite direction of the leading strand synthesis that is catalyzed by conventional DNA polymerase $\delta$ (Dutta and Bell, 1997). Interestingly, the GANP primase activity measured *in vitro* is regulated by the phosphorylation state at $Ser^{502}$ that is close to the primase homology domain

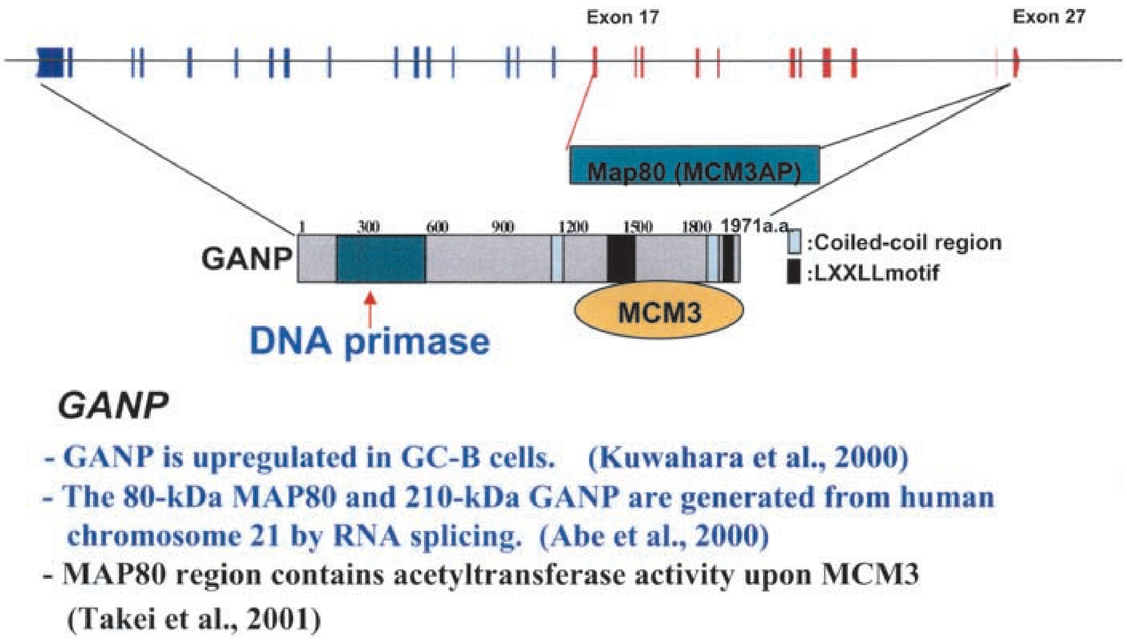

FIGURE 1 Structure of *ganp* gene. The *ganp* and *Map80* genes are created by an alternative splicing mechanism of the *ganp*/*Map80* gene transcripts. The exons from 1st to 27th are used for *ganp* and the exons from 17th to 27th are used for *Map80* gene. Map80 region is associated with MCM3 and has a MCM3-acetyltransferase activity (Map80/MCMAP). There is a primase domain in the amino-terminal region. Other potential motifs are also shown in the schema. Several important functions of GANP molecule are listed as below.

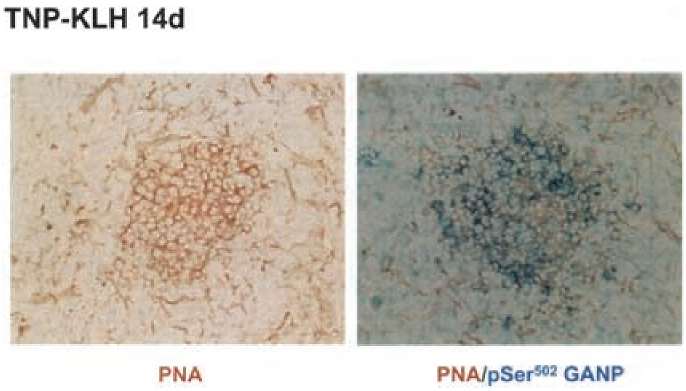

FIGURE 2 GANP expression in GC-B cells. Spleen section was prepared at 14 days after immunization of C57BL/6 mouse with TD-Ag (TNP-KLH) in complete Freund's adjuvant. The cryosection was processed and incubated with PNA-biotin/streptavidin-peroxidase and rat anti-phosphoserine of $Ser^{502}$-GANP mAb/goat anti-rat IgG Ab conjugated with alkaline phosphatase. The left panel shows a single color image with PNA staining of GC area, and the right panel shows double staining profile of the GC area together with the mAb for the primase-active GANP expression. The result suggests that GC-B cells express GANP primase activity.

(Kuwahara *et al.*, 2001). The specific mAb to the phorphorylated $Ser^{502}$ of GANP showed that the phosphorylation is induced by the stimulation with anti-CD40 cross-linking *in vitro* or the stimulation with TD-Ag *in vivo*. Thus, GANP is a second-type eukaryotic RNA-primase that is expressed and up-regulated in GC-B cells (Fig. 2).

The information regarding GANP suggested that GANP has domains participating in DNA-replication or cell proliferation. Therefore, we hypothesized that GANP is required in the regulation of proliferation of Ag-driven B cells in association with either one of the differentiation processes of GC-B cells described above.

### GANP Function in Eukaryotic Cells

To determine the function of GANP in the immune response associated with GCs, we tried to prepare the *ganp*-gene knockout mouse by homologous recombination method, however, the mice were embryonic lethal before generation of lymphoid cells in the fetal liver (unpublished data). This information indicated that GANP expression is essential not only for B cell development but also for the mammalian cell development at the embryonic stage. While it was not determined in detail, the embryos had multi-organ deformities as early as E12. We prepared the mouse embryonic fibroblast (MEF) cells by conventional method and demonstrated that the cell proliferation was not severely impaired in *ganp* gene-knockout MEFs in comparison with the wild-type MEF cells (unpublished data). However, when the *ganp* gene was over-expressed in human B cell lines, the DNA contents in the transfectants increased more than the tetraploid contents by flow cytometric analysis (Kuwahara *et al.*, 2001). The results may accord with the expected function of GANP, which is a positive regulator for DNA replication in eukaryotic cells. Expression of GANP might assist the DNA replication when the GC-B cells or the embryonic cells need to proliferate at an exceedingly rapid pace for the clonal expansion within a limited period of time. We recently prepared the conditionally gene-knockout mice that lack GANP expression in $CD19^{+}$ B cells using Cre-loxP system (Kuwahara *et al.*, submitted for publication). Flow cytometric analysis of the mutant B cells ($ganp^{-/-}$ B cells) showed that maturation of B lineage cells was normal during the early B cell development from the bone marrow to the spleen, however, the $ganp^{-/-}$ B cells showed the apparent alteration of the normal development upon immunization with Ags. GC-B cells in the mutant ($ganp^{-/-}$ B) mice did not show the maturation from the $sIgM^{+}sIgD^{-}$ B cells to $sIgM^{low}sIgD^{high}$ B cells, suggesting that GANP is involved in the differentiation of B cells upon immunization with TD-Ag.

### Function of GANP in Humoral Immune Response

Since GANP is selectively involved in GC-B cells, we compared the immune responses of the mutant mice to T cell-independent Ag (TI-Ag) and TD-Ag *in vivo*. Ag-specific response was measured by ELISA and the formation of GC region was analyzed by the immunohistochemical analysis. The response to TI-Ag was not impaired in mutant mice but the response to TD-Ag was impaired by the study using ELISA and the immunohistochemical analysis of detecting GC formation. The results demonstrated that GANP is required for formation of GCs during the response to TD-Ags *in vivo*. GANP is a MCM3-associated RNA/DNA-primase, whose activity might be involved in the formation of GCs or the differentiation of B cells in the GC area.

### DISCUSSION

We carried out the experiments to characterize the function of GANP in GC formation during humoral immune response. The molecular structure is highly suggestive for its involvement in DNA replication and the data also suggested that DNA replication is affected by the over-expression of GANP molecule in B cells. The *ganp* gene-knockout mice demonstrated the evidences suggesting that GANP is also involved in the differentiation processes of B cells associated with TD-Ag immunization. In mutant (ganp$^{-/-}$ B cells) mice, B cell maturation in the peripheral lymphoid tissues was affected by the observation after stimulation with TD-Ag *in vivo*. The humoral response was not impaired upon immunization with TI-Ag but was impaired in response to TD-Ag. These results implied that GANP function is not merely necessary to support the rapid proliferation of B cells but is also involved in the differentiation of B cells during the formation of GCs after immunization with TD-Ag. For the function of GANP upon differentiation of GC-B cells, there are many questions to be answered. What is a role of GANP at the centrocyte stage? Does GANP play some role in regulation from centroblast to centrocyte transition? How GANP RNA-primase activity is involved in B cell differentiation? Is the existence of GANP necessary for somatic V-region hypermutation? Does GANP participate for class switching?

Somatic hypermutation of immunoglobulin V-region is detected in the centroblast at the dark zone of GCs, which suggested that DNA-replication is required for the introduction of V-region hypermutation (DNA-replication coupled mechanism). Recent evidences, however, suggested that the introduction of V-region somatic hypermutation is rather dependent on the transcription at the somatic hypermutation V-region (SHV region) and RNA-editing mechanism is critical for the addition of mutations (RNA-transcription coupled mechanism). The RNA primers generated by GANP would be mostly template-independent, thus creating the initial chain as template independent nucleotide at the region of lagging strand synthesis. If the primers generated by GANP primase show higher affinity with one of the DNA polymerases involved in DNA repair mechanism, they might cause a higher incidence of DNA breaks or the mismatch repair during DNA replication. Alternatively, RNA primers might be engaged in the process of RNA-transcription coupled function by unknown mechanism. The third possibility is the association of GANP molecule with various functional molecules in B cells for cell proliferation and differentiation. It may recruit a molecule that is necessary for GC-B cells either in the cell cycle control or the gene transcription. Our attempt to determine a molecule associated with GANP, demonstrated that a phosphatase component named G5PR is associated with GANP and may cause the alteration of phosphorylation states during B cell differentiation in GCs (Kono *et al.*, 2002). The 210-kDa protein of GANP may be a molecule generating a multiple protein complex with GANP, ori-complex components, phosphatases and presumably, with other components such as HSP90 as a chaperone component, and the associated components of various nuclear transcription factors. It is necessary to demonstrate the components associated with GANP/MCM3 and to study the regulatory mechanism in the B cell proliferation and differentiation in GCs.

### *Acknowledgements*

This work was supported by a Grant-in-aid from The Ministry of Education, Science, Culture, Sports and Technology in Japan and the Uehara Memorial Foundation.

### *References*

Abe, E., Kuwahara, K., Yoshida, M., Suzuki, M., Terasaki, H., Matsuo, Y., Takahashi, E. and Sakaguchi, N. (2000) “Structure, expression, and chromosomal localization of the human gene encoding a germinal center-associated nuclear protein (GANP) that associates with MCM3 involved in the initiation of DNA replication”, *Gene* **255**, 219–227.

Arezi, B. and Kuchta, R.D. (2000) “Eukaryotic DNA primase”, *Trends in Biochemical Science* **25**, 572–576.

Dutta, A. and Bell, S.P. (1997) “Initiation of DNA replication in eukaryotic cells”, *Annual Review of Cellular and Developmental Biology* **13**, 293–332.

Ishimi, Y. (1997) “A DNA helicase activity is associated with an MCM4, -6, and -7 protein complex”, *Journal of Biological Chemistry* **272**, 24508–24513.

Kelsoe, G. (1996) “Life and death in germinal centers (redux)”, *Immunity* **47**, 107–111.

Kono, Y., Maeda, K., Kuwahara, K., Yamamoto, H., Miyamoto, E., Yonezawa, K., Takagi, K. and Sakaguchi, N. (2002) “MCM3-binding GANP DNA-primase is associated with a novel phosphatase component G5PR”, *Genes to Cells* **7**, 821–834.

Kuwahara, K., Yoshida, M., Kondo, E., Sakata, A., Watanabe, Y., Abe, E., Kouno, Y., Tomiyasu, S., Fujimura, S., Tokuhisa, T., Kimura, H., Ezaki, T. and Sakaguchi, N. (2000) “A novel nuclear phosphoprotein, GANP, is up-regulated in centrocytes of the germinal center and associated with MCM3, a protein essential for DNA replication”, *Blood* **95**, 2321–2328.

Kuwahara, K., Tomiyasu, S., Fujimura, S., Nomura, K., Xing, Y., Nishiyama, N., Ogawa, M., Imajoh-Ohmi, S., Izuta, S. and Sakaguchi, N. (2001) “Germinal center-associated nuclear protein (GANP) has a phosphorylation-dependent DNA-primase activity that is up-regulated in germinal center regions”, *Proceedings of the National Academy of Science USA* **98**, 10279–10283.

MacLennan, I.C.M. (1994) “Germinal centers”, *Annual Review of Immunology* **12**, 117–139.

Rajewsky, K. (1996) “Clonal selection and learning in the antibody system”, *Nature* **381**, 751–758.

Takei, Y. and Tsujimoto, G. (1998) “Identification of a novel MCM3-associated protein that facilitates MCM3 nuclear localization”, *Journal of Biological Chemistry* **273**, 22177–22180.

Takei, Y., Swietlik, M., Tanoue, A., Tsujimoto, G., Kouzarides, T. and Laskey, R. (2001) “MCM3AP, a novel acetyltransferase that acetylates replication protein MCM3”, *EMBO Report* **2**, 119–123.

Waga, S. and Stillman, B. (1998) “The DNA replication fork in eukaryotic cells”, *Annual Review of Biochemistry* **67**, 721–751.

